# Simultaneous RNA-Seq Analysis of a Mixed Transcriptome of Rice and Blast Fungus Interaction

**DOI:** 10.1371/journal.pone.0049423

**Published:** 2012-11-06

**Authors:** Yoshihiro Kawahara, Youko Oono, Hiroyuki Kanamori, Takashi Matsumoto, Takeshi Itoh, Eiichi Minami

**Affiliations:** 1 Agrogenomics Research Center, National Institute of Agrobiological Sciences, Tsukuba, Ibaraki, Japan; 2 Genetically Modified Organism Research Center, National Institute of Agrobiological Sciences, Tsukuba, Ibaraki, Japan; Seoul National University, Republic of Korea

## Abstract

A filamentous fungus, *Magnaporthe oryzae*, is a causal agent of rice blast disease, which is one of the most serious diseases affecting cultivated rice, *Oryza sativa*. However, the molecular mechanisms underlying both rice defense and fungal attack are not yet fully understood. Extensive past studies have characterized many infection-responsive genes in the pathogen and host plant, separately. To understand the plant-pathogen interaction comprehensively, it is valuable to monitor the gene expression profiles of both interacting organisms simultaneously in the same infected plant tissue. Although the host-pathogen interaction during the initial infection stage is important for the establishment of infection, the detection of fungal gene expression in infected leaves at the stage has been difficult because very few numbers of fungal cells are present. Using the emerging RNA-Seq technique, which has a wide dynamic range for expression analyses, we analyzed the mixed transcriptome of rice and blast fungus in infected leaves at 24 hours post-inoculation, which is the point when the primary infection hyphae penetrate leaf epidermal cells. We demonstrated that our method detected the gene expression of both the host plant and pathogen simultaneously in the same infected leaf blades in natural infection conditions without any artificial treatments. The upregulation of 240 fungal transcripts encoding putative secreted proteins was observed, suggesting that these candidates of fungal effector genes may play important roles in initial infection processes. The upregulation of transcripts encoding glycosyl hydrolases, cutinases and LysM domain-containing proteins were observed in the blast fungus, whereas pathogenesis-related and phytoalexin biosynthetic genes were upregulated in rice. Furthermore, more drastic changes in expression were observed in the incompatible interactions compared with the compatible ones in both rice and blast fungus at this stage. Our mixed transcriptome analysis is useful for the simultaneous elucidation of the tactics of host plant defense and pathogen attack.

## Introduction

Rice is one of the world's most important food crops, and it feeds over 50% of the world's population. A map-based, finished quality sequence of *Oryza sativa* L. ssp. *japonica* cv. Nipponbare was determined by the International Rice Genome Sequencing Project (IRGSP) in 2005 [1]. The curated genome annotation has continued to be provided by the Rice Annotation Project (RAP) and is available in the RAP-DB [2]. A filamentous fungus, *Magnaporthe oryzae*, which is the causal agent of blast disease in rice, is the most serious pathogen affecting rice worldwide. Infection by the rice blast fungus causes annual yield losses averaging upwards of 30% [3]. The genome of the rice blast fungus strain “70–15” was determined in 2005 [4]. Automated gene predictions and annotations were performed by the Broad Institute, and the latest version of the data (MG6) is available on the website http://www.broadinstitute.org/annotation/genome/magnaporthe_grisea/MultiHome.html. In addition, National Institute of Agrobiological Sciences (NIAS) constructed cDNA libraries of *M. oryzae* strain Guy11 and performed gene annotation based on the mate-pair EST sequences of the cDNA clones and ab initio gene prediction [5]. The availability of reference genome sequences and gene annotation enabled us to use *O. sativa* and *M. oryzae* as models to study the molecular interactions between the host plant and its pathogen.

To understand the molecular mechanisms of the establishment of the infection of rice with blast fungus, extensive studies have characterized many rice and fungal genes that are involved in plant defense and pathogen attack [3], [6]–[8]. Several studies have conducted genome-wide expression profile analyses, revealing the entire rice defense system at the early infection stage using the oligonucleotide microarray technique [9], [10]. The fungal genes that are responsible for spore germination and appressorium formation have been relatively well characterized because these states are easily induced on a hydrophobic surface that mimics the rice leaf surface and by the addition of exogenous cAMP to spore suspensions [11], [12]. Recently, a microarray study compared gene expression levels in *M. oryzae* mycelium grown under different stress conditions with those of the fungus invading the plant [13]. Another study constructed a cDNA library of expressed genes in the infected rice leaves [14]. However, although these studies investigated in planta fungal gene expression, they analyzed only a subset of fungal genes or an expressed gene catalog in which expression levels were not provided. Furthermore, they focused on gene expression levels at a relatively late infection stage (3–10 days post-inoculation (dpi)), which was too late to investigate the early infection processes that are important during its establishment. Because only a small number of plant cells are invaded, and it is difficult to detect the gene expression levels of the fungal genes in the infected tissue, the ascertainment of the genome-wide gene expression profile and elucidation of the molecular mechanisms of blast fungus invasion at the early infection stage in infected rice leaves have remained poorly understood.

The expression profiles of both the rice and blast fungus genes in a spatially and temporally equivalent biological sample could help us to understand the molecular mechanisms of both rice defense and blast fungus infection strategies simultaneously. To date, a few studies have analyzed the genome-wide expression profiles of both the rice and blast fungus genes simultaneously in the same infected sample. Matsumura *et al*. [15] analyzed infected leaves at 10 dpi to obtain a sufficient amount of fungal RNA for the SuperSAGE analysis. As previously mentioned, this is too late of a stage to investigate the molecular interaction that is involved in the establishment of infection. Mosquera *et al*. [16] examined both rice and fungal gene expression profiles at a relatively early infection stage (36 hours post-inoculation (hpi)) using oligonucleotide microarray technology. However, the detection of fungal gene expression in infected tissues using microarrays requires tough mechanical sample manipulations, such as the fluorescent labeling of invaded fungal cells and chopped, infected rice cells by laser microdissection. These sample manipulations may have had outside effects on the host plant and pathogen cells, and it is difficult to observed infection-responsive reactions in a natural state. Furthermore, in that study, infected leaf sheaths were used as the host plant samples, but plant cells on the surfaces of leaf blades are invaded by blast fungus in nature. Thus, it is necessary to obtain the responsive expression profiles of the infected leaf blades to understand the true molecular interactions between rice and blast fungus.

Emerging massively parallel sequencing techniques enable the rapid acquisition of huge amounts of genomic or transcriptomic sequence data at relatively low costs [17]. To date, microarray techniques have been predominantly used for gene expression analyses particularly for well-studied model organisms for which typically high-quality gene annotation data were available. Compared with microarrays, RNA-Seq is known to have a wider dynamic range, higher technical reproducibility, and provide a better estimate of absolute expression levels [18], [19]. Although two NGS methodologies, RNA-Seq and high throughput-SuperSAGE (HT-SuperSAGE), provide very similar levels of sensitivity from the points of view of the sequencing cost and accuracies of the measured expression levels, HT-SuperSAGE is much more cost-effective [12]. However, the expression profiles of unannotated transcripts cannot be analyzed by microarrays because the probes on microarray chips are designed based on annotation data. HT-SuperSAGE also requires gene structure information to convert tag counts to corresponding transcript expression levels and does not provide entire gene structures. However, RNA-Seq can measure the expression levels of all transcripts without prior knowledge because entire RNA molecules can be sequenced, and all sequence information from the transcribed regions can be obtained. Recently, several transcript structure prediction methods that are based on reference genomes and RNA-Seq data have been developed, and unannotated transcript structures can be constructed with their expression levels using the programs [20]–[22].

To elucidate the comprehensive gene expression profiles for both the host plant and its fungal pathogen simultaneously, we developed a workflow to analyze the mixed transcriptome of rice and blast fungus that was obtained from infected rice leaf blades using RNA-Seq. Here, we report that this method can detect both rice and in planta fungal gene expression, and large numbers of rice and fungal transcripts are responding to infection at 24 hpi. At this stage, approximately 50% of the intact appressorium succeeded in the primarily penetration, while less than 1% of those extended hyphae into more than one cell of epidermis of leaf sheath (unpublished data). Many pathogenesis-related genes, WRKY type transcription factors and phytoalexin biosynthetic genes showed strong responsive expression in rice. For blast fungus, approximately 38% of the responsive transcripts encoded putative secretory proteins, suggesting that they are good candidate fungal effectors that are involved in attacking a host plant. Using compatible (Ina86-137) and incompatible (P91-15B) fungal strains as pathogens, we revealed the differential expression profiles of the compatible and incompatible interactions and observed that the responsive gene expression was more drastic in the incompatible interaction. Using the RNA-Seq method, we observed the transcriptional activity on the genome at the nucleotide level, and information describing all transcribed regions and expression profiles before and after the infection are available for both rice and blast fungus from the Plant-Pathogen Mixed RNA-Seq Database http://anise.dna.affrc.go.jp/ppmix/.

## Results and Discussion

### RNA-Seq analysis of mixed transcriptome samples

**Figure 1:**
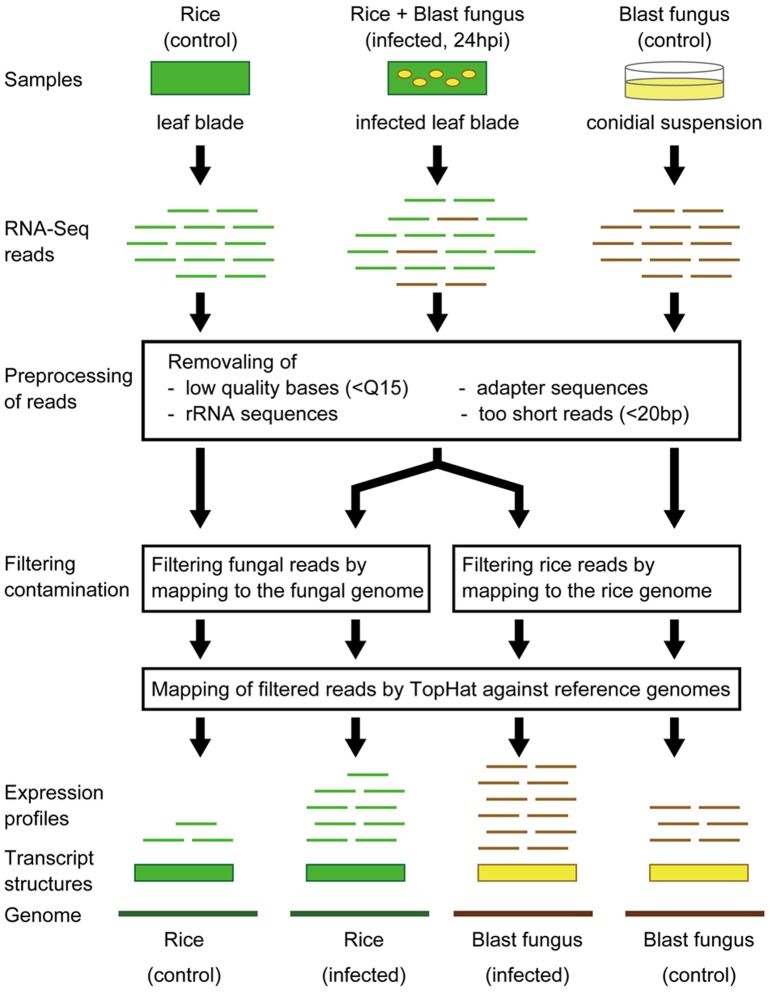
Schematic representation of RNA-seq analysis of mixed transcriptome obtained from blast fungus-infected rice leaves. First, mRNA were extracted from the *Oryza sativa* ssp. *japonica* cv. Nipponbare (Pia) rice leaf blades 24 hours after water treatment (rice, control) and inoculation (rice+blast fungus, infected, 24hpi), and also from conidial suspensions of the compatible and incompatible blast strains (blast fungus, control). RNA-Seq were conducted for each sample using the illumine GAIIx sequencer. In the preprocessing of reads, low quality bases, adapter sequences, rRNA sequences and too short reads (<20 bp) were removed. For the rice analysis, all of the preprocessed reads were mapped to the fungal genome to filter out contaminated fungal reads. For the fungal analysis, contaminated rice reads were removed by mapping all of the reads against the rice genome. Finally, all of the filtered reads were mapped to the reference genomes by TopHat and transcript structures are predicted by Cufflinks. For each rice and fungal transcript, expression levels were estimated using the numbers of uniquely mapped reads to the transcript structures.

To investigate the comprehensive gene expression profiles of the host plant *Oryza sativa* L. ssp. *japonica* cv. Nipponbare and its pathogen *Magnaporthe oryzae* simultaneously at the initial infection stage, we analyzed the mixed transcriptome of rice and blast fungus that was sampled from the fourth rice leaf blades at 24 hpi. As described above, hyphae from less than 1% of the intact appressoria invaded more than one cell of epidermis of leaf sheath at this stage (unpublished data). Kankanala *et al*. [23] reported that the infected cells are no longer viable after hyphae moved to adjacent cells, indicating that the host and fungal responses including gene expression are different in primarily infected cells from those in secondarily infected cells. By these reasons, we chose this stage to minimize the level of transcripts from the secondarily infected cells. Therefore, the profiles of gene expression in this study would be originated largely from the infectious hyphae located in the single host cells. To reveal any differences in the responsive expression profiles between the compatible and incompatible plant-pathogen interactions, we used compatible Ina86-137 (COM) and incompatible P91-15B (INC) fungal strains against *Oryza sativa* L. ssp. *japonica* cv. Nipponbare (NPB) carrying the rice blast resistance gene Pia. Here, we performed illumina RNA-Seq on the rice and fungal control samples before infection (NPB, Ina86-137 and P91-15B) and the infected, mixed transcriptome samples (NPB+Ina86-137 and NPB+P91-15B) with a 76-cycle single-end protocol (DRA000542). Two biological replicates were included in each mixed transcriptome sample. After the preprocessing of the reads, we obtained 42, 35, 9, 15 and 11 million single-end reads (read lengths ranging from 20 to 76 bp) for the control samples (NPB, Ina86-137 and P91-15B) and the infected mixed transcriptome samples (NPB+Ina86-137 and NPB+P91-15B), respectively (Table 1). To obtain the expression profiles and predict gene structures, we mapped the preprocessed RNA-Seq reads against each reference genome using the Bowtie, TopHat and Cufflinks programs [20]–[22] (Figure 1).

**Table 1:** Mapping results of RNA-Seq reads.

	Control			Infected mixed transcriptome			
Samples	NPB^a^	Ina86-137 (COM)	P91-15B (INC)	NPB+Ina86-137 (COM)		NPB+P91-15B (INC)	
**Preprocessed reads**	8,898,395	14,826,277	11,305,038	41,625,682		34,696,199	
**Genome** b	IRGSPb5	MG6	MG6	IRGSPb5	MG6	IRGSPb5	MG6
**All mapped reads (%)**	5,396,461 (60.6)	12,544,801 (84.6)	9,844,205 (87.1)	25,585,194 (61.6)	52,268 (0.1)	21,656,843 (62.4)	61,049 (0.2)
**- Uniquely mapped reads (%)**	5,094,614 (57.3)	11,196,817 (75.5)	6,683,476 (59.1)	24,252,843 (58.3)	28.297 (0.1)	20,563,795 (59.3)	31,49 (0.1)
**Unmapped (%)**	3,501,934 (39.4)	2,281,476 (15.4)	1,460,833 (12.9)	15,988,220 (38.4)		12,978,307 (37.4)	

a NPB: control sample of *Oryza sativa* ssp. *japonica* cv. Nipponbare.

b Reference genome sequences used for mapping; IRGSPb5: *Oryza sativa* ssp. *japonica* cv. Nipponbare IRGSP build 5 genome, MG6: Magnaporthe grisea 70–15 release 6 genome.

**Figure 2:**
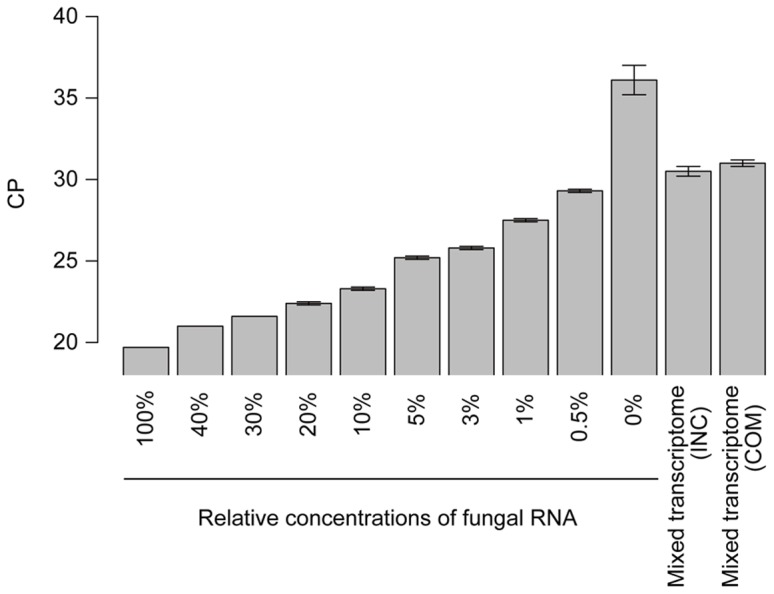
quantitative Real-Time RT-PCR experiments to assess fungal RNA contents in mixed transcriptome samples. The presence of fungal RNA in the mixed transcriptome samples (COM: compatible, INC: incompatible interactions) was examined by qRT-PCR using artificial mixed transcriptome samples at several fungal RNA concentrations (0, 0.5, 1, 3, 5, 10, 20, 30, 40 and 100%). Crossing point (CP) values represent the point at which the amount of the amplified fungal actin reached a fixed threshold. High CP values indicate low abundance of fungal RNAs. The means and standard errors of CP values of three technical replicates are shown for each sample.

At the initial stage (24 hpi) of rice and blast fungus infection, because most plant cells in the leaf blades have not yet encountered the fungal invasion, the amount of fungal cells that are present in the plant cells is expected to be very low compared with that of the rice leaf cells. Therefore, the detection of fungal transcripts in a mixed transcriptome that has been isolated from infected leaves is expected to be very difficult. The mapping results of the RNA-Seq reads could provide rough estimates of the ratios of rice and fungal RNA concentrations in the mixed transcriptome samples because the number of RNA-Seq reads should be correlated with the amount of RNA molecules in a sample. Indeed, for the mixed transcriptome, 61.5–62.4% of the preprocessed reads mapped to the host rice genome but only 0.1–0.2% mapped to the fungal genome. Based on the mapping results, the ratios of the numbers of mapped reads to the rice and fungal genomes are 490∶1 (25,585,194∶52,268) for NPB+Ina86-137 and 355∶1 (21,656,843∶61,049) for NPB+P91-15B (Table 1), indicating an estimated frequency of fungal transcripts in the mixed transcriptome of 0.2–0.3%. The fungal RNA concentrations in the mixed transcriptome were assessed by quantitative Real-Time RT-PCR of artificial mixed transcriptome samples with several concentration gradients (Figure 2). As a result, the concentration of the fungal RNAs in the samples were estimated to be between 0–0.5%. These frequencies were consistent with estimates that were based on the mapping rates (0.2–0.3%). Fungal RNA concentrations will increase throughout the course of infection, and the detection of fungal RNAs in infected samples should be easier in the later infection stages. Still, a large amount of reads could not be mapped to the reference genomes (12.9–39.4%). There could be several reasons for the unmapped reads, such as sequencing errors, failure to remove the adapter, the presence of contaminated sequences that could not be detected in the filtering processes and an incomplete of the reference genome. In particular, for the fungal transcriptome, because we used the fungal strains (Ina86-137 and P91-15B) that differ from the reference strain (70–15), the unmapped reads may be derived from strain-specific or highly divergent transcribed regions.

### Estimation of expression profiles and detection of infection-responsive rice and fungal transcripts at the initial infection stage

In principle, the number of RNA-Seq reads should be correlated with the amount of RNA molecules in a sample. By using the normalized number of reads overlapped with the transcribed regions, we can estimate the expression levels of each transcript. Here, we calculated the normalized expression levels (RPKM: reads per kilobase exon model per million mapped reads) of each rice and fungal transcript using uniquely mapped reads onto the genomes [24]. Two biological replicates were available for the mixed transcriptome samples. To evaluate the reproducibility and biological variations of the mixed transcriptome analysis, we first calculated the RPKMs of the annotated rice and fungal transcripts for each biological replicate separately and compared them between replicates. The resulting Pearson's correlation coefficients (*R*) were significantly high (*R* = 0.81–0.99; *P*<0.001) between the replicates for both the rice and blast fungus (Figure S1). Compared with the correlation coefficients for the rice transcripts (*R* = 0.89–0.99; *P*<0.001), those for the blast transcripts (*R* = 0.81–0.83; *P*<0.001) were relatively lower. The lower correlations were due to the smaller number of mapped reads for the blast fungus. To increase the accuracies of the measured expression levels for further analyses, data from two biological replicates were merged, and RPKM values were calculated based on the merged dataset.

**Figure 3:**
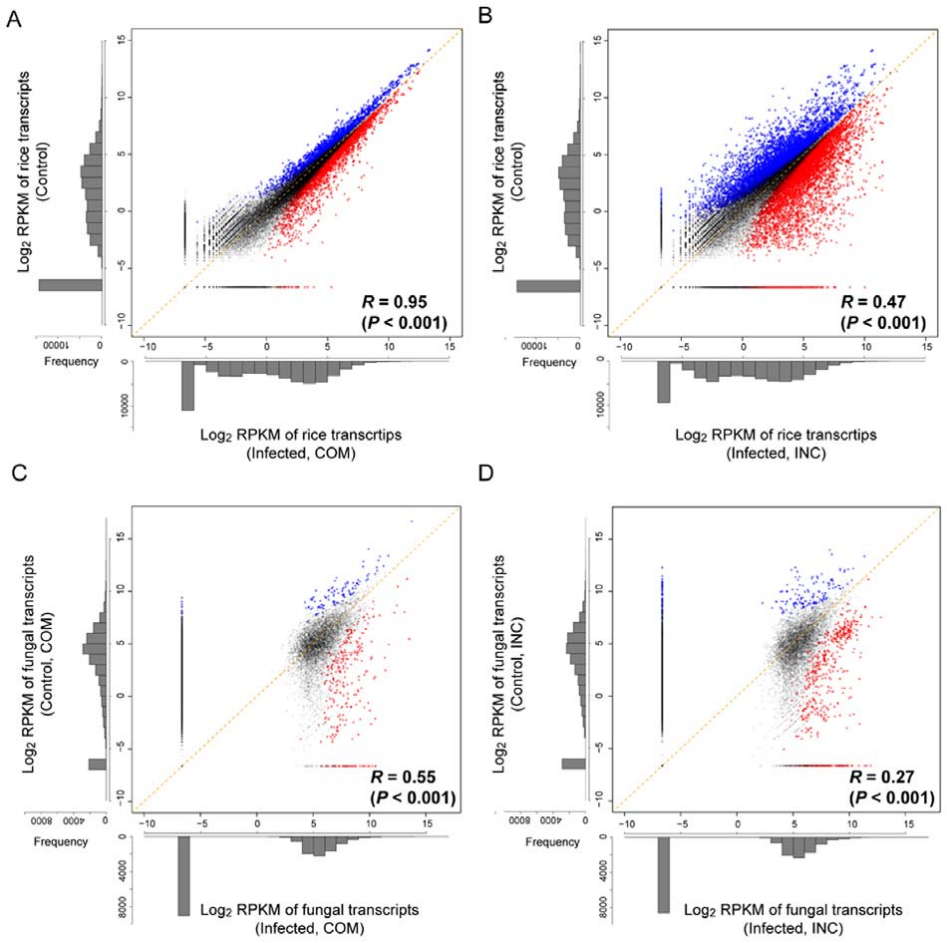
Distributions of expression levels for rice and fungal annotated transcripts. The distributions of the expression levels (log_2_ RPKMs) are shown for rice (A, B) and blast fungus (C, D). For rice, (A) the control (NPB) and infected (NPB+Ina86-137) samples in the compatible interaction and (B) the control (NPB) and infected (NPB+P91-15B) samples in the incompatible interaction are compared. For blast fungus, (C) the control (Ina86-137) and infected (NPB+Ina86-137) samples in the compatible interaction and (D) the control (P91-15B) and infected (NPB+P91-15B) samples in the incompatible interaction are compared. Significantly differentially expressed transcripts between the control and infected conditions are plotted in colors (red: upregulated, blue: downregulated). Pearson's correlation coefficients (*R* ^2^) between replicates are presented.

The estimations of the expression levels revealed that known rice transcripts have mean (±SD) expression levels (RPKMs) of 24.3±233.4, 23.1±163.3 21.1±111.6 for the control (NPB), compatible (NPB+Ina86-137) and incompatible (NPB+P91-15B) interaction samples, respectively (Figure 3). The mean values (±SD) of the RPKMs for the fungal transcripts were 57.2±837.5, 52.9±253.2, 48.0±225.4 and 49.6±161.7 for the compatible (Ina86-137), incompatible (P91-15B) control, compatible (NPB+Ina86-137) and incompatible (NPB+P91-15B) interaction samples, respectively (Figure 3). For the mixed transcriptome samples, the minimum RPKM value among the fungal annotated transcripts was 2.59, which was much larger than that of rice (0.01), suggesting that lowly expressed transcripts may be missed to detect for the blast fungus. Such low levels of in planta fungal gene expression could not be detected by the oligonucleotide microarray due to the lower detection sensitivity; however, in principle, RNA-Seq can detect any level of expression if sufficient sequencing data is available. Substantial progress in sequencing technology will overcome the issues of sequencing throughput and the sensitivity of RNA-Seq analyses in the near future. However, although there were many undetectable transcripts with low expression levels, the expression levels of most genes (60.2%; 10,142/16,856 fungal annotated transcripts) were detected (RPKM>0) in this RNA-Seq analysis.

To examine whether the two fungal strains (Ina86-137 and P91-15B) and the reference strain (70–15) are similar at the molecular level, we estimated the nucleotide differences between the strains using uniquely mapped RNA-Seq reads on the reference genome. As a result, the number of nucleotide substitution was estimated to be 0.00018 for both Ina86-137 and P91-15B. Therefore, all the three strains are quite similar at the transcribed sequence level. Furthermore, the distributions of RPKM values were almost identical between the two fungal strains in the conidial condition (Figure S2). Taken together, these results suggest that the evolutionary divergence of the fungal strains did not lead to biased estimations of gene expression levels.

The overall trends of changes in expression levels that are caused by fungal infection could be regarded as correlated to the RPKM distributions of the control and infected samples. For rice, a higher correlation of expression levels between the control and compatible interaction (NPB vs. NPB+Ina86-137; *R* = 0.95; *P*<0.001) was observed compared with that between the control and incompatible interaction (NPB vs. NPB+P91-15B; *R* = 0.47; *P*<0.001) (Figure 3A–B). The correlation of the expression levels of the fungal transcripts indicated that more stable and similar expression profiles existed between the two different fungal strains in the conidial condition (Ina86-137 vs. P91-15B; *R* = 0.65; *P*<0.001) compared with those that were infected (NPB+Ina86-137 vs. NPB+P91-15B; *R* = 0.59; *P*<0.001). In the comparisons between the control and infected samples, the compatible interaction (Ina86-137 vs. NPB+Ina86-137; *R* = 0.55; *P*<0.001) showed a higher correlation than the incompatible interaction (P91-15B vs. NPB+P91-15B; *R* = 0.27; *P*<0.001) (Figure 3C–D). Taken together, these results indicate that in both the rice and blast fungus, the changes in expression levels that are caused by rice-fungal infection are greater in the incompatible than the compatible interaction. This suggests that the responsive reactions that are involved in the plant defense and pathogen attack are more active in the incompatible interaction.

Compared with the microarray and SuperSAGE techniques, the biggest advantage of the RNA-Seq technique is that it can predict exon-intron structures and their expression levels concurrently without any prior knowledge. Using the Cufflinks program, we predicted novel unannotated rice and fungal transcripts that did not overlap with any annotated transcripts. As a result, 6,223 and 2,341 unannotated transcribed regions of 100 bp or longer in length were predicted in the rice and blast fungus genomes, respectively. We calculated expression levels for each unannotated transcript using the same method as that for known transcripts. The mean (±SD) expression levels of the unannotated rice transcripts were 3.2±17.4, 3.1±20.2 and 3.0±16.5 for the control, compatible and incompatible interaction samples, respectively. For the unannotated fungal transcripts, the mean expression levels were 33.8±718.9, 108.3±2525.0, 135.3±3250.2 and 98.2±2247.2 for the compatible, incompatible control, compatible and incompatible interaction samples, respectively. The unannotated rice transcripts may have had relatively lower expression levels and may be specifically expressed in certain conditions, because the rice annotation is quite enriched, and the majority of commonly expressed transcribed regions have already been discovered. The large variations in the expression levels of the fungal unannotated transcripts were partially due to the same reasons as those of rice. Furthermore, highly expressed rRNA may still be contaminating the predictions because blast fungus rRNA sequences were not fully available, and yeast rRNA sequences were used in the filtering process instead.

**Figure 4:**
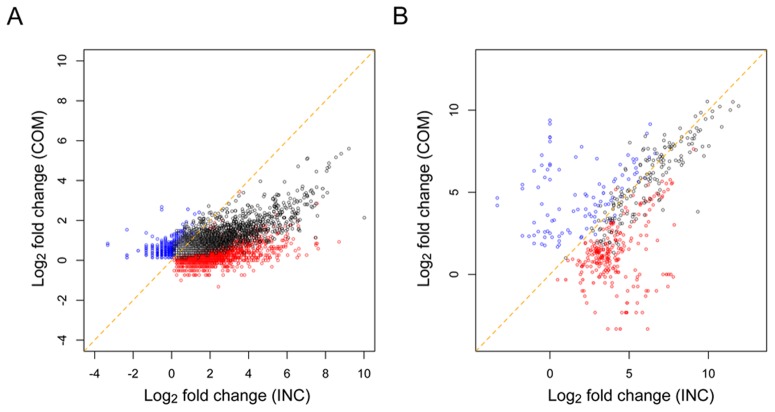
Distribution of fold-changes of rice and fungal significantly upregulated transcripts. The fold-changes (log_2_) of the significantly upregulated transcripts in the compatible and incompatible interactions are plotted for (A) rice and (B) blast fungus. The colors represent the types of interactions in which the upregulations occurred (black: Common, blue: COM-specific, red: INC-specific).

To find the differentially expressed rice and fungal transcripts between the control and infected conditions, we comprehensively compared the expression levels of each transcript between the two conditions and conducted a statistical test, the G-test, for each comparison with a 0.1% false discovery rate (FDR) [25]. As a result, 15,616 (30.0%) and 872 (5.2%) transcripts showed significant differential expression in the annotated rice and fungal transcripts, respectively (Table 2; all differentially expressed annotated transcripts are listed in Table S1, S2). From the Cufflinks-predicted unannotated transcripts, 432 (6.9%) rice and 17 (0.7%) fungal transcripts also showed differential expression (Table 2; all differentially expressed unannotated transcripts are listed in Table S3, S4). The infection-responsive differential expression of 5,311 (34.0%) and 234 (26.8%) annotated, and 63 (14.6%) and 6 (35.3%) unannotated transcripts of rice and blast fungus, respectively, were commonly observed in the compatible and incompatible interactions (“Common”), and the remaining showed different responsive expression profiles between the two interactions (“COM-specific” and “INC-specific”). Furthermore, larger numbers of transcripts responded to the infection in the incompatible compared with the compatible interaction, and this pattern was commonly observed for both the rice and blast fungus. The fold-changes of the upregulated rice and fungal transcripts were greater in the incompatible interaction even though there were significant commonly upregulated transcripts in both the compatible and incompatible interactions (Figure 4). These observations support the aforementioned drastic infection-responsive reactions of the rice and blast fungus in the incompatible interaction. The differential responses of rice defense-related transcripts to the infection of compatible and incompatible fungal strains has been observed using microarray and northern blot analyses [26]–[28]. However, this is the first report to show the differential response of in planta fungal gene expression between compatible and incompatible interactions at the initial infection stage.

**Table 2:** Number of significant infection-responsive rice and fungal transcripts.

	Annotated		Cufflinks-predicted unannotated	
	Upregulated	Downregulated	Upregulated	Downregulated
**Rice** a				
Common	3,310	2,001	26	37
COM-specific	1,329	131	23	9
INC-specific	3,902	4,943	161	176
**Blast fungus** b				
Common	163	71	6	0
COM-specific	108	41	5	0
INC-specific	359	130	2	4

a Numbers of all annotated and Cufflinks-predicted rice transcripts were 52,053 and 6,223, respectively.

b Numbers of all annotated and Cufflinks-predicted fungal transcripts were 16,856 and 2,341, respectively.

**Figure 5:**
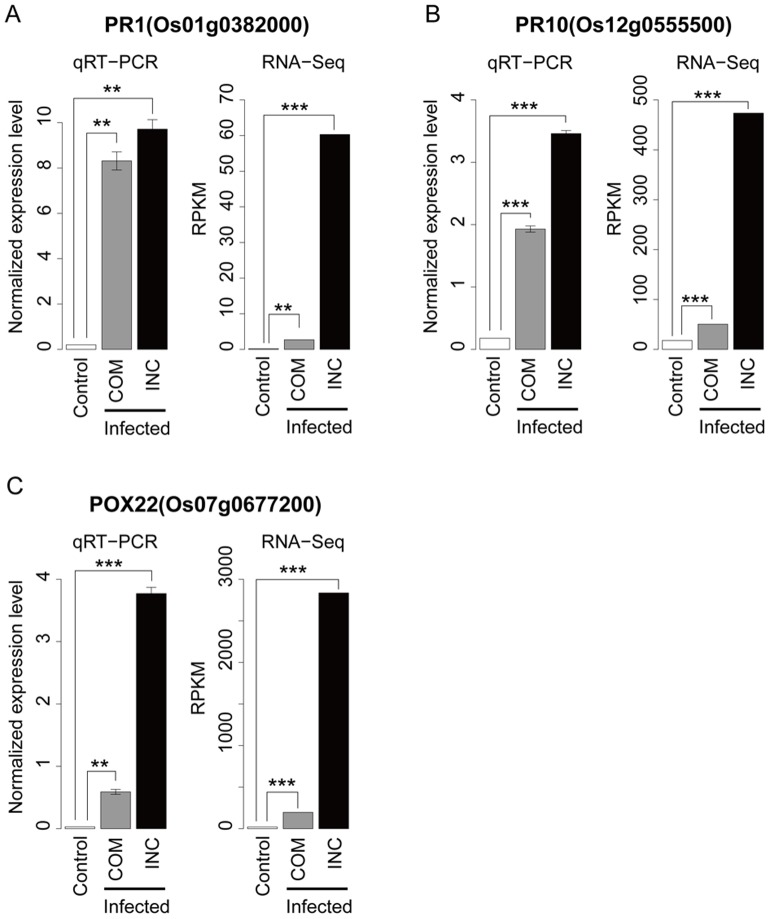
Confirmation of differential expression of known infection-responsive rice genes by qRT-PCR and RNA-Seq. The expression profiles of known infection-responsive rice genes, (A) PR1, (B) PR10 and (C) POX22, were examined by both qRT-PCR and RNA-Seq techniques. For the qRT-PCR data, the mean±standard errors for the three replicates are represented. The statistically significant differential gene expression between the control (Cont) and infected (Inf) samples for the compatible (COM) and incompatible (INC) interactions was tested by the Student's *t*-test and G-test for qRT-PRC and RNA-Seq, respectively. The asterisks show the statistical significances (*: *P*<0.05, **: *P*<0.01 and ***: *P*<0.001).

**Figure 6:**
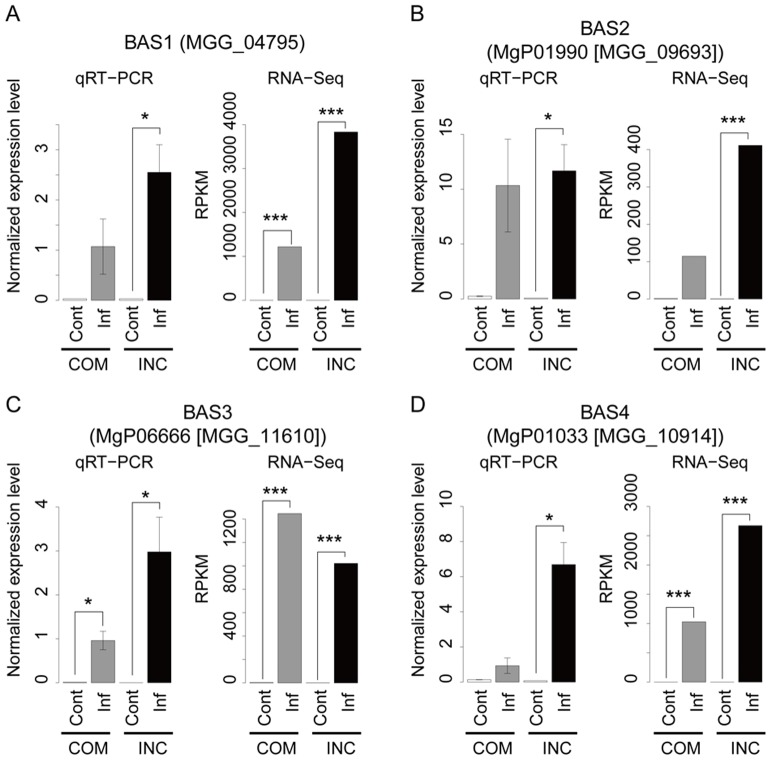
Confirmation of differential expression of known infection-responsive fungal genes by qRT-PCR and RNA-Seq. The expression profiles of known infection-responsive fungal genes, biotrophy-associated secreted (BAS) proteins (A) 1, (B) 2, (C) 3 and (D) 4, were examined by both qRT-PCR and RNA-Seq techniques. For the qRT-PCR data, the mean±standard errors for the three replicates are represented. The statistically significant differential gene expression between the control (Cont) and infected (Inf) samples for the compatible (COM) and incompatible (INC) interactions were tested by the Student's *t*-test and G-test for qRT-PRC and RNA-Seq, respectively. The asterisks show the statistical significances (*: *P*<0.05, **:*P* <0.01 and ***: *P*<0.001).

To examine whether the previously reported infection-responsive rice and fungal transcripts were detected as differentially expressed in our mixed transcriptome RNA-Seq analysis, the expression levels of several known responsive transcripts were measured by qRT-PCR using gene-specific primers (Table S5). The rice pathogenesis-related proteins PR1 and PR10 were chosen because they have been shown to display strong induction during infection with blast fungus [29], [30]. One of rice peroxidase genes, POX22, which was also reported to show induced expression during the infection of *Xanthomonas oryzae* and *Magnaporthe grisea*, was also used for validation [26], [31]. As a result of both the RNA-Seq and qRT-PCR analyses, PR1, PR10 and POX22 were characterized to be differentially expressed at the initial infection stage (Figure 5). For PR1, no significant difference was observed between the control and compatible interaction at the 0.1% FDR level. However, at the 1% FDR level, PR1 showed significant differential expression. Furthermore, significantly stronger responsive upregulations were observed in the incompatible compared with the compatible interaction for all three genes (FDR<0.1%). To confirm the differential expression of well-known fungal-responsive transcripts, we chose members of the BAS family of genes (BAS1, BAS2, BAS3 and BAS4), which have been reported to show responsive expression patterns and encode biotrophy-associated secretory proteins [16]. The upregulation of these fungal genes was confirmed by both RNA-Seq and qRT-PCR (Figure 6). However, the stronger responsive upregulations in the incompatible compared with the compatible interaction as seen in the rice-responsive genes were not observed for the fungal BAS genes. These results suggest that the degree of differential responses between the compatible and incompatible interactions differed among genes and also proved that our mixed transcriptome analysis can detect the infection-responsive expressions that has been reported in past studies in both rice and blast fungus even at the initial infection stage.

### Responsive expression of rice defense-related genes at the initial infection stage

We detected the infection responsive up-regulation of 8,541 annotated and 210 Cufflinks-predicted unannotated transcripts. Of those, 23 rice-annotated genes with functional annotations showed greater than 150-fold upregulations in their expression levels in either the compatible or incompatible interactions (highly upregulated rice genes; Table 3). Of those, 18 genes showed upregulation in both the compatible and incompatible interactions, and the remaining were incompatible-specific upregulated genes. However, compatible-specific, highly elevated upregulations were not observed. Furthermore, even for the common highly upregulated genes, the fold changes for the incompatible interaction (average fold change: 280.7) were approximately 22 times greater than those for the compatible interaction (average fold change: 12.9). These results indicate that the rice defense responses against fungal infection were stronger in the incompatible interaction at the initial infection stage. During the infection of *Xanthomonas oryzae* pv. *oryzae*, it has been reported that rice peroxidase genes show faster responsive expression in the incompatible compared with the compatible interactions [26]. Another study, which used Agilent rice DNA chips (G4138A, Agilent Technologies) with time-series samples at 24 and 48 hpi, reported that the peaks of the responsive expression of rice genes against fungal infection in the compatible interaction were delayed compared with those in the incompatible interaction [27]. However, these studies examined only a limited number of genes. The findings in our comprehensive RNA-Seq analysis indicate that differential inductions of the expression of infection-responsive genes between the compatible and incompatible interactions were commonly observed at the genome-wide level at the initial infection stage.

**Table 3:** Highly upregulated (fold change≥150) rice genes.

Gene ID	Fold change		Annotation	Secreted
	COM	INC		
**Common**				
Os03g0797500	4.4	1036.3	Similar to Indole-3-glycerol phosphate lyase	No
Os01g0615100	48.5	594.1	Similar to Substilin/chymotrypsin-like inhibitor (Proteinase inhibitor)	No
Os06g0530600	40.7	449.5	VQ motif containing protein	No
Os01g0615050	35.9	372.1	Proteinase inhibitor I13, potato inhibitor I family protein	No
Os01g0940700	24.9	274.4	Similar to Glucan endo-1,3-β-glucosidase GII precursor	Yes
Os12g0555000	10.5	273.0	Similar to Probenazole-inducible protein PBZ1	No
Os12g0628600	22.3	266.6	Similar to Thaumatin-like pathogenesis-related protein 3 precursor	Yes
Os12g0555200	9.2	233.5	Similar to Probenazole-inducible protein PBZ1	No
Os08g0509100	12.1	231.9	Similar to Lipoxygenase	No
Os12g0437800	4.7	191.0	Similar to MPI (maize protease inhibitor)	No
Os01g0816100	12.7	185.9	Similar to NAC domain protein [OsNAC4]	No
Os06g0649000	21.9	184.2	Similar to WRKY transcription factor 28	No
Os10g0527800	7.2	181.2	Similar to Tau class Glutathione S-transferase protein 3	No
Os11g0530600	2.2	180.0	Similar to Chalcone synthase C2	No
Os05g0427400	6.7	172.4	Similar to Phenylalanine ammonia-lyase	No
Os12g0448900	10.2	164.1	Similar to Pathogen-inducible alpha-dioxygenase	No
Os03g0663500	6.5	154.2	Similar to Osmotin precursor	Yes
Os01g0955100	7.8	151.5	Similar to Avr9/Cf-9 rapidly elicited protein 57	No
**INC-specific**				
Os07g0526400	1.9	416.8	Polyketide synthase, type III domain containing protein	No
Os07g0526600	1.5	196.5	Alpha/beta hydrolase fold-3 domain containing protein	No
Os09g0417600	1.8	188.9	WRKY transcription factor 76	No
Os03g0830500	1.7	180.9	Similar to PGPS/D12	Yes
Os06g0569500	2.2	177.6	Similar to Ent-kaurene oxidase 1 [OsKOL2]	Yes

Well-curated functional annotations of rice transcripts are available in the RAP-DB and were used to investigate the functional features of infection-responsive upregulated transcripts. As a result, we found a large number of pathogenesis-related (PR) protein family genes showing upregulation in the initial infection stage (Table S6A, B, C, D, E). The term PR protein is defined as a protein that accumulates during pathogen infection. The plant PR proteins were well characterized and classified into the PR1-17 families [32]. We found 21 upregulated genes encoding proteinase inhibitors belonging to the PR1 family that were known to participate in the growth inhibition of a variety of pathogenic bacterial and fungal strains and thought to be useful for the development of antimicrobial agents [33]. Chitinases are classified into the PR3, 4, 8 and 11 families and are able to bind and hydrolyze chitin in the cell walls of plant pathogens. The expression levels of 24 chitinases were upregulated and most of them were predicted to be putative secretory proteins in our analysis, suggesting that they were secreted outside of cells to degrade the cell walls of pathogens. Glycosyl hydrolases (GHs), which are enzymes that catalyze the hydrolysis of the glycosidic bonds between sugars and other moieties, can be classified into more than 100 families [34]. In particular, β-1,3-glucanases (GH17) catalyze the endo-type hydrolytic cleavage of the 1,3-β-D-glucosidic linkages in β-1,3-glucans and are referred to as pathogenesis-related proteins belonging to the PR2 family [35]. Peroxidase (*POX*) genes, which are classified into the PR9 family, are known to be induced by pathogen infection, suggesting that they are defense-related proteins [26]. The infection-responsive upregulation of 23 GH17 protein-coding genes, including β-1,3-glucanases and 40 peroxidase genes, were detected in our analysis. The upregulation of these PR genes indicates that the degradation of the cell wall components (glucans, chitin and proteins) of pathogens is an important defense reaction in rice against fungal pathogens at the initial infection stage.

Major rice diterpene phytoalexins (PAs) are known to be low-molecular-weight compounds that are produced in response to pathogen attack. To date, the accumulation of the PAs after fungal infection has been reported for momilactone A, oryzalexin E, oryzalexin S and phytocassanes A-E in rice. The increased expression levels of genes that are involved in PA biosynthesis were also observed [36]. Here, we found that many genes that are involved in the PA biosynthesis were upregulated at the initial infection stage. The expression levels of OsCPS2 (Os02g0571100) and OsKSL7 (Os02g0570400), which are involved in phytocassane A-E synthesis, were upregulated in the incompatible interaction. Moreover, OsCPS4 (Os04g0178300) and OsKSL4 (Os04g0179700), which are involved in the biosynthesis of momilactone A and B also showed incompatible-specific upregulation. OsKSL10 (Os12g0491800) and OsKSL8 (Os11g0474800), which are involved in the biosynthesis of oryzalexin A-F and S, showed upregulation in both the compatible and incompatible interactions. However, the fold changes of OsKSL10 and OsKSL8 expression were larger in the incompatible compared with the compatible interaction. Recently, more abundant and rapid phytoalexin accumulations in the resistant compared with the susceptible plant have been reported at 2 dpi [36]. Our results suggested that, when the same rice cultivar, Nipponbare (Pia), is used in our analysis, the stronger induction of the expression of rice phytoalexin biosynthetic genes are observed in the infection of the incompatible fungal strain compared with that of the compatible one at the initial infection stage.

Plants are able to recognize potential microbial pathogens through pathogen-associated molecular patterns (PAMPs) by host sensors, which are known as pattern-recognition receptors (PRRs) that initiate a series of defense responses called PAMP-triggered immunity (PTI) [6]. Mitogen-activated protein kinase (MAPK) cascades are known to play important roles in mediating PAMP signals and also in plant stress responses [37], [38]. Several MAPK and MAPK kinase (MAPKK) genes have been characterized in the response of plants to fungal infection [39]. We found that the expression of OsMPK6 (Os06g0154500), which is known to be essential for the chitin elicitor-induced biosynthesis of diterpenoid phytoalexins, was specifically induced in the incompatible interaction. The inductions of the expressions of OsMPK3 (Os03g0285800) and OsMKK4 (Os02g0787300) were observed in both the compatible and incompatible interactions at the initial infection stage. The hypersensitive response (HR) is a common plant immune response against the infection of pathogens and is a type of programmed cell death. We found that the transcription factor OsNAC4 (Os01g0816100), which plays a role in the initiation of hypersensitive cell death in rice [40], was also upregulated at the initial infection stage. Other transcription factors, including the WRKY family genes, have been suggested to play roles in controlling the transcription of defense-related genes through the W-box in their promoters, which is a key cis-element in defense-related transcriptional regulation [10], [41], [42]. The upregulated rice transcripts included 41 genes that were annotated as WRKY transcription factors. In particular, the expression levels of WRKY transcription factors 19, 26, 28, 45, 62 and 76 were highly upregulated (>50 fold-change), suggesting that they may play important roles as key regulators in the rice defense response against the infection of blast fungus at the initial infection stage.

One of the biggest advantages of the RNA-Seq technique is that it predicts novel, unannotated transcript structures and their expression levels. The Cufflinks-predicted unannotated rice transcripts included 210 that were upregulated at the initial infection stage. Eight of those transcripts encoded putative protein kinase family proteins (Rice_CUFF.34181.1, Rice_CUFF.34181.2, Rice_CUFF.2283.1, Rice_CUFF.2282.1, Rice_CUFF.7414.1, Rice_CUFF.23507.1, Rice_CUFF.28691.1 and Rice_CUFF.7415.1), two were NB-ARC domains that are thought to be involved in disease resistance, including the transcripts Rice_CUFF.6912.1 and Rice_CUFF.26220.2, and one was an oxidoreductase domain containing transcript Rice_CUFF.28752.2. As described above, our analysis could detect the infection-responsive expression of a large number of genes that are thought to be involved in plant defense against fungal pathogens. Therefore, these unannotated, upregulated transcripts may have important functions in rice defense against pathogen infection. These results strongly suggest that even for the well-annotated rice genome, RNA-Seq is a powerful tool for the detection of unannotated transcripts and the enrichment of functional annotations for not only unannotated but also annotated transcripts.

### Responsive expression of fungal effector candidates at the initial infection stage

The infection-responsive upregulation of 630 and 13 fungal annotated and unannotated transcripts were detected, respectively. Of those 35 fungal genes having any functional annotations, showed a greater than 150-fold upregulation in their expression levels in either the compatible or incompatible interactions (highly upregulated fungal genes; Table 4). These transcripts may play important roles during pathogen attack in the host plant at the initial infection stage.

**Table 4:** Highly upregulated (fold change≥150) fungal genes.

	Fold change			
Gene ID	COM	INC	Annotation	Secreted
**Common**				
MGG_04795	1219.6	3833.4	BAS1	Yes
MgP01033 (MGG_10914)	1030.3	2672.9	BAS4	Yes
MgP00943 (MGG_08526)	1476.0	976.6	Calpain family cysteine protease	No
MgP03906 (MGG_04732)	220.8	791.9	acidic mammalian chitinase	Yes
MgP06666 (MGG_11610)	310.7	546.7	BAS3	Yes
MgP11598 (MGG_08390)	361.3	150.4	dipeptidyl aminopeptidase/acylaminoacyl peptidase	No
MgP05141 (MGG_02201)	222.8	203.2	endothiapepsin	Yes
MgP08240 (MGG_15040)	340.3	64.7	cytochrome P450 3A9	Yes
MgP07935 (MGG_02778)	219.1	183.5	Hydrophobic surface binding protein A	Yes
MgP06218 (MGG_00715)	260.4	32.9	Similar to Glucose-repressible gene protein (P22151)	No
MgP00511 (MGG_09640)	156.2	131.2	alpha-amylase 1	Yes
MgP05331 (MGG_09085)	201.6	73.2	malic acid transport protein	No
MgP03306 (MGG_05575)	165.5	107.6	Hydrophobic surface binding protein A	Yes
MgP03986 (MGG_05555)	88.2	182.3	xenobiotic compound monooxygenase	No
MgP11608 (MGG_08379)	200.7	60.8	cytochrome P450	Yes
MgP11266 (MGG_12337)	190.3	58.4	MAS3 protein	Yes
MgP01421 (MGG_03988)	74.0	162.7	Collagen triple helix repeat (20 copies)	Yes
**COM-specific**				
MgP00850 (MGG_08498)	575.8	1.0	isotrichodermin C-15 hydroxylase	Yes
MgP04809 (MGG_02329)	218.1	4.0	isotrichodermin C-15 hydroxylase	No
MgP11597 (MGG_08391)	154.0	30.5	Zinc-binding dehydrogenase	No
**INC-specific**				
MgP02232 (MGG_10097)	38.4	715.3	intracellular hyphae protein 1 (LysM domain)	Yes
MgP10026 (MGG_08957)	199.0	534.7	Similar to OTU domain-containing protein 1 (Q9CUB6)	No
MgP01990 (MGG_09693)	53.4	278.4	BAS2	Yes
MgP05133 (MGG_02212)	72.0	256.1	Hydrophobic surface binding protein A	Yes
MgP08325 (MGG_10237)	0.9	288.2	Similar to Ice-structuring glycoprotein (P24856)	Yes
MgP08339 (MGG_12505)	46.0	204.0	alpha-ketoglutarate-dependent taurine dioxygenase	No
MgP11630 (MGG_08356)	54.3	192.6	polysaccharide deacetylase family protein	No
MgP08310 (MGG_10254)	8.1	229.8	taurine catabolism dioxygenase TauD	No
MgP01674 (MGG_03764)	0.9	220.5	salicylate hydroxylase	No
MgP01122 (MGG_04257)	0.9	219.5	secretory lipase family protein	Yes
MgP06107 (MGG_00824)	35.9	175.1	phenylacetone monooxygenase	No
MgP07676 (MGG_03036)	46.4	163.1	NmrA-like family	No
MgP07648 (MGG_14179)	29.5	172.1	Ubiquitin 3 binding protein But2 C-terminal domain	Ys
MgP09137 (MGG_03421)	1.0	181.4	Phosphotransferase enzyme family	No
MgP01647 (MGG_03793)	1.0	164.9	2,3-dihydroxybenzoic acid decarboxylase	No

The genes involved in appressorium development should be included in the upregulated genes, because we compared the fungal gene expression profiles in germinated conidia and infected leaves at 24 hpi. To examine how many upregulated genes were related to appressorium development, we compared our data with 1,696 genes that were upregulated in the process of appressorium development in a previous study [12]. As a result, 29.6% of the upregulated transcripts detected in our RNA-Seq analysis were also upregulated during appressorium development, suggesting that these genes play important roles in the appressorium development.

Rice blast effectors, which are secreted from biotrophic-invasive hyphae at the plant-pathogen interface and suppress the innate immune response, are known to play important roles in the establishment of fungal infection. Through the prediction of signal sequences and the cellular localization of each fungal transcript, we found that a significantly larger number of upregulated transcripts (38.1% = 240/630) encoded putative secreted proteins that may be potential candidates of fungal effectors compared with the non-responsive transcripts (14.3% = 2,285/15,974) (Table 5). In particular, more than half of the highly upregulated genes (54.3% = 19/35) encoded putative secreted proteins. This suggests that the upregulation of secreted protein-coding genes is a remarkable feature of fungal attack at the initial infection stage. We also found 69 other highly upregulated fungal genes having no functional annotation in the MgNEST-DB and Broad Magnaporthe grisea Database, which were expected to encode putative secretory genes, suggesting that the functional annotation of the fungal genome was still insufficient and that these genes are good candidates for novel fungal effectors (Table S7).

**Table 5:** Numbers of fungal putative secretory protein-coding genes for each differential expression category.

Category	Number of putative secreted proteins (%)	Significance a
Blast fungus, upregulation		
- Common	94 (57.7)	**
- COM-specific	51 (47.2)	**
- INC-specific	95 (26.5)	**
Blast fungus, downregulation		
- Common	22 (31.0)	**
- COM-specific	7 (17.1)	-
- INC-specific	19 (14.6)	-

a Significance of enrichment compared with non-differentially expressed transcripts was assessed by Fisher's exact test: **: *P*<0.001, *: *P*<0.01.

Two LysM domain-containing intracellular hyphae protein-coding genes, Slp1 (MgP02232 [MGG_10097]) and Slp2 (MgP09192 [MGG_03468]), which were annotated as putative secretory proteins, were found to be upregulated. Recently, it was proposed that fungal LysM effectors play roles in the sequestration of chitin oligosaccharides to suppress PAMP-triggered host immunity [43], [44]. Indeed, Slp1 showed binding activity with chitin and was able to suppress the chitin-induced plant immune response [45]. In addition, four MAS3 (*Magnaporthe* appressoria-specific) protein-coding genes (MgP06228 [MGG_00703], MgP11266 [MGG_12337], MgP01185 [MGG_04202], MgA02141 [MGG_00703]), which are essential for the pathogenicity of *M. oryzae* and highly expressed in the appressoria [46], were up-regulated.

A cuticle, which is composed of an insoluble polymeric structural compound, cutin, protects plant leaf surfaces. One of the functions of the cuticle layer is to act as a physical barrier that resists penetration by fungal pathogens. Plant pathogenic fungi produce extracellular degradative enzymes that play important roles in pathogenesis. They include cutinase, which hydrolyses cutin, facilitating fungal penetration through the cuticle [47]. Our analysis found that two cutinase genes, MgP04456 [MGG_02393] and MgP00673 [MGG_14095], were upregulated, suggesting that they may play a role in the degradation of plant cuticles at the initial infection stage. Furthermore, five fungal chitinases, MgP03906 [MGG_04732], MgP03585 [MGG_05533], MgA00835 [MGG_06594], MgP06906 [MGG_00086] and MgP02656 [MGG_06594] were also upregulated. It has been suggested that in filamentous fungi, chitinases may act during hyphal growth and autolysis [48]. Therefore, the upregulation of these five fungal chitinases may be involved in the control of hyphal growth in planta during the infection.

In a large-scale secretome study using two-dimensional gel electrophoresis coupled with mass spectrometry analysis under nitrogen starvation conditions, which are similar to the conditions that are present during fungal infection, the authors reported that the identified proteins were mainly cell wall hydrolase enzymes, protein and lipid hydrolases and reactive-oxygen-species-detoxifying proteins [49]. In our analysis, two phenylacetone monooxygenases (MgA00533 [MGG_09323] and MgP06107 [MGG_00824]), three ent-kaurene oxidases (MgP11603 [MGG_08385], MgP06649 [MGG_00300] and MgP01608 [MGG_03834]), two galactose oxidases (MgP08908 [MGG_10878] and MgP08366 [MGG_12681]), a hydroxyquinol 1,2-dioxygenase (MgA00497 [MGG_03773]) and a 12-oxophytodienoate reductase (MgA00148 [MGG_10583]), which are enzymes that belong to the family of oxidoreductases, showed highly up-regulated expression profiles. Furthermore, in addition to the plant defense reaction, many transcripts encoding fungal glycosyl hydrolase family proteins were also highly up-regulated in blast fungus at the initial infection stage, including an endoglucanase belonging to the glycoside hydrolase family 61 (MgA03201 [MGG_06069]), an endo-1,4-β-xylanase (MgP00996 [MGG_14243, MGG_14244]) and an alpha-mannosidase (MgP06908 [MGG_00084]). It is known that fungi constantly produce several glycosyl hydrolase enzymes that degrade cell wall polysaccharides during cell division, growth and morphogenesis [50]. The expression of glycoside hydrolase proteins was shown to contribute to the degradation of host cell wall polysaccharides, and the soluble oligosaccharides that are produced by glycoside hydrolase digestion were transported to the inside of the fungal cell and metabolized [51]. A large-scale microarray study found that a group of genes encoding enzymes that are involved in cell wall degradation, glucan mobilization and cell wall glycoprotein processing were upregulated in appressorium induction and maturation [11]. Taken together, these results suggest that glycosyl hydrolase family proteins play important roles in host cell wall degradation and appressorium formation at the initial infection stage. In addition, in our RNA-Seq analysis, the upregulation of glycosyl hydrolase-encoding transcripts was commonly observed in rice and blast fungus, suggesting that the enzymes that are involved in cell wall degradation play important roles in both plant defense and pathogen attack.

There were six transcripts encoding HsbA (hydrophobic surface binding protein A) domain-containing proteins (MgP07935 [MGG_02778], MgP11632 [MGG_08354], MgP03306 [MGG_05575], MgP05133 [MGG_02212], MgP00995 [MGG_11009] and MgP07089 [MGG_09460]) in the fungal responsive transcripts. It was reported that *Aspergillus oryzae* HsbA adsorbed to hydrophobic PBSA surfaces in the presence of NaCl or CaCl_2_ and promoted polybutylen succinate-co-adipate (PBSA) degradation via the CutL1 polyesterase [52]. Our results suggested that *Magnaporthe oryzae* may function in the biodegradation of polymers and be used for fungal biodegradation.

Of all upregulated fungal transcripts, 278 (44.1%) had no meaningful functional annotation in the current databases. These transcripts may play some roles during the initial infection stage of rice with blast fungus. Furthermore, 13 Cufflinks-predicted fungal unannotated transcripts were upregulated. Of those, two transcripts were annotated as putative secreted protein-coding transcripts in our analysis, suggesting that they may be candidate novel fungal effectors.

### Conclusions

**Figure 7:**
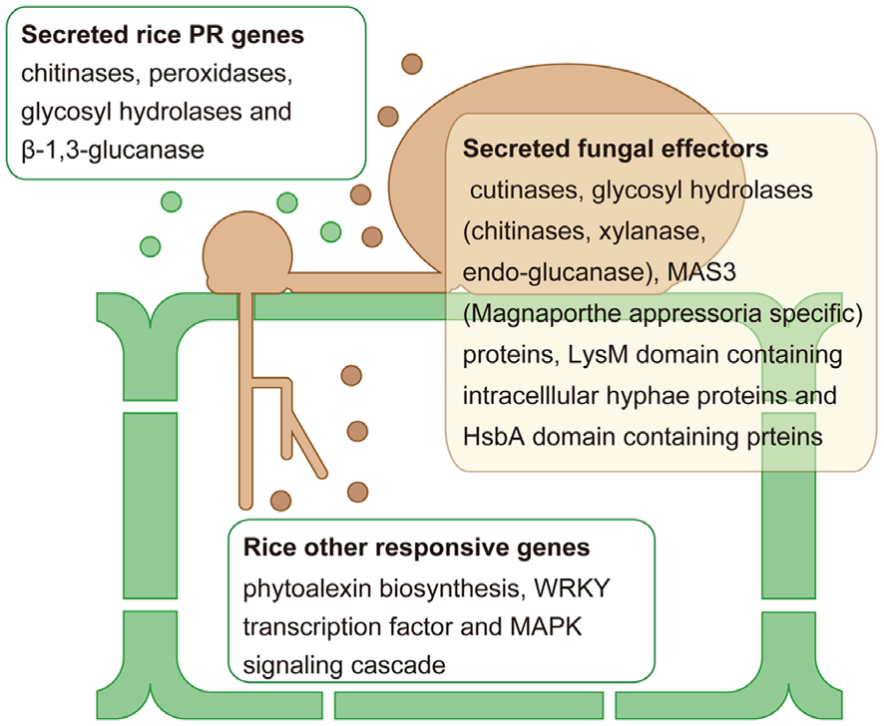
Overview of rice plant defense and blast fungus pathogen attack at the initial infection stage.

Using RNA-Seq, we have revealed the expression profiles of both rice and blast fungus simultaneously in rice-infected leaf blades at the initial infection stage (24 hpi). This analysis provided comprehensive infection-responsive expression profiles for both rice and fungal transcripts that may interact at precise moments and locations. We found that 16,048 rice and 889 fungal transcripts, including 432 and 17 unannotated ones, respectively, showed infection-responsive expression. These findings provide us with knowledge regarding the molecular basis of the interaction of rice and blast fungus at the initial infection stage (Figure 7). In addition, we found unannotated infection-responsive genes that may play roles in plant defense and pathogen attack. Using two different types of fungal strains, we revealed that the differential responsive expression patterns between the compatible and incompatible interactions and more drastic responsive reactions in the incompatible interaction were common features of both rice and blast fungus at the initial infection stage. To reveal the responsive expression profile more precisely through the infection stages, additional time-course samples will be needed. Furthermore, this RNA-Seq analysis has provided information regarding transcriptional activity at the nucleotide level, unannotated novel transcript structures and expression profiles at the initial infection stage for both rice and blast fungus using the Plant-Pathogen Mixed RNA-Seq Database (http://anise.dna.affrc.go.jp/ppmix/). This information is valuable, particularly for the blast fungus research community, because the fungal genome still has relatively poor functional annotations. Our research provides a powerful tool for the comprehensive mixed transcriptome analysis of various combinations of host plants and pathogens.

## Materials and Methods

### Preparation of control and infected samples for host plants and pathogens

*Oryza sativa* L. ssp. *japonica* cv. Nipponbare carrying the rice blast resistance gene, Pia, was used as the host plants. The rice blast fungus *Magnaporthe oryzae* field isolates, P91-15B (race 001.0) and Ina86-137 (race 007.0, MAFF 101511), were used as the compatible and incompatible pathogens against Nipponbare (NPB), respectively. The fungal strains were cultured on oatmeal agar plates at 26°C. Seedlings of rice were grown hydroponically in a nutrient solution for 3 weeks as described previously [53]. The fourth leaves of intact plants were sprayed with the conidial suspensions. The fourth leaves of the inoculated plants were dissected at 24 hpi and used as infected samples (NPB+P91-15B and NPB+Ina86-137). For the rice control sample (NPB), the fourth leaves were dissected at 24 hpi with distilled and sterile water. All samples were frozen in liquid nitrogen immediately after collection and stored at -80°C. For the blast control samples, conidia of both fungal isolates were incubated for 1 hour at room temperature to allow germination followed by RNA extraction. At this time point, most of the conidia (76–87%) were germinated.

### RNA extraction, mRNA-seq library construction and sequencing

For all samples, total RNAs were extracted using TRIzol® reagent (Life Technologies, Rockville, MD, USA). Total mRNAs were isolated by oligo(dT) selection using Dynal® magnetic beads (Invitrogen). Double-stranded cDNA molecules were generated using random hexadeoxynucleotide primers and then sequenced using the illumina (San Diego, CA) mRNA-Seq single-end protocol on a Genome Analyzer IIx with 76 cycles.

### Preprocessing and mapping of illumina reads

In total, 11,106,935, 11,718,807, 14,695,841, 41,610,361 and 50,942,135 single-end reads of 76 bp in length were obtained from the NPB, P91-15B, Ina86-137, NPB+P91-15B and NPB+Ina86-137 samples, respectively. All of the primary sequencing data can be found in the DDBJ Sequence Read Archive (DRA) under the accession number DRA000542. Using an in-house program, low quality bases (<Q15) were trimmed out from both the 5′- and 3′- ends of reads until a stretch of 3 bp or more of high quality (≥Q15) bases appeared. Contaminated illumina adapter sequences (5′ P-GATCGGAAGAGCGGTTCAGCAGGAATGCCGAG) were also trimmed out using a python script “cutadapt” (ver. 0.9.4) [54]. Rice and fungal reads derived from ribosomal RNAs (rRNAs) were removed by the mapping of all reads against rice rRNA sequences in the RAP-DB and yeast rRNA sequences (RDN18-1, RDN18-2, RDN25-1, RDN25-2, RDN37-1, RDN37-2, RDN5-1, RDN5-2, RDN5-3, RDN5-4, RDN5-5, RDN5-6, RDN58-1 and RDN58-2) in the Saccharomyces Genome Database (SGD), respectively, using the Bowtie program (ver. 0.12.7) with the default parameters. Additionally, reads that were too short in length (<20 bp) were discarded after the aforementioned processes. At the final step of read preprocessing, to avoid the contamination of reads from the interaction partners (which were rice for blast fungus and vice versa), we mapped all of the reads against the reference genome of the interaction partner (filtering contamination step in Figure 1). Next, the remaining unmapped non-blast and non-rice reads were used to examine the expression profiles of rice and blast fungus, respectively.

### Measurement of gene expression levels and detection of novel unannotated transcripts

Annotated rice transcripts (52,053 transcripts) on the IRGSP build5 reference genome were downloaded from the RAP-DB as of June 1^st^, 2011. For blast fungus, two different annotation datasets on the *Magnaporthe grisea* Assembly release 6 were downloaded from the Magnaporthe grisea Database at the Broad Institute [4] and Magnaporthe grisea NIAS EST Database (MgNEST-DB) at National Institute of Agrobiological Sciences [5]. To construct a unified fungal transcript dataset, we merged all of the transcripts in the MgNEST-DB (16,257 transcripts) with those in the Magnaporthe grisea Database that did not overlap with any of the MgNEST-DB transcripts (599 transcripts).

A series of programs, including Bowtie, TopHat (ver. 1.3.0) and Cufflinks (ver. 1.0.3), were used to map reads against reference genomes and predict transcript structures based on the RNA-Seq data. Cufflinks-predicted transcripts that did not overlap with any other annotated transcripts were used as the novel unannotated transcripts. The gene expression levels for each annotated and novel unannotated transcript were estimated as the number of reads per kilobase of exon model per million mapped reads (RPKM) using only uniquely mapped reads in exonic regions. The significantly differentially expressed transcripts between the control and infected samples were detected using the G-test at the 0.1% significance level. The FDR [25] was used to maintain the significance level at 0.1%. Fold changes of expression levels between samples were calculated after adding one to each RPKM value to avoid division by zero.

### Functional annotation for annotated and novel unannotated transcripts

To infer the molecular functions of the rice and fungal novel unannotated transcripts, we performed homology searches against the protein databases (Swiss-Prot entries in UniProt release 2011_1 [55], and validated and reviewed protein entries in RefSeq release 45 [56]) using the BLASTx (ver. 2.2.24) program with the following parameters: -e 1e-2. Because the unified fungal transcripts were derived from two different data sources, we also conducted a homology search for all of the fungal annotated transcripts to conduct an annotation using the same criteria. Putative protein-coding sequences for each novel unannotated transcript were predicted by the getorf program in the EMBOSS package (ver. 5.0.0) [57]. The longest ORF sequences (≥30aa) were used for further analyses. To assign further functional information to the rice and fungal protein-coding transcripts, we conducted a functional domain search using the Pfam database release 25.0 [58] and the “pfam_scan.pl” script, and then gene ontology terms were assigned to each transcripts using the pfam2go conversion table that is provided on the Gene Ontology website (http://www.geneontology.org/). To identify the transcripts encoding putative secretory proteins, signal peptides were predicted by SignalP 3.0 [59], and cellular localizations were predicted by TargetP 1.1 [60] and WoLFPSORT ver. 0.2 [61] for each transcript. Proteins that were predicted as secretory proteins by SignalP and also predicted as secretory pathways or extracellular localized proteins by TargetP and WoLFPSORT, respectively, were considered to be putative secretory proteins.

### Estimation of the fungal RNA concentrations in the mixed transcriptome samples by quantitative Real-Time RT-PCR (qRT-PCR)

cDNA was synthesized using 1 µg of the total RNA of the mixed transcriptome samples from infected leaves and the artificial mixed transcriptome samples (0, 0.5, 1, 3, 5, 10, 20, 30, 40 and 100% fungal RNA) by the SuperScript® III First-Strand Synthesis System (Invitrogen, Carlsbad, CA, USA) after DNase I (TaKaRa, Shiga, Japan) treatment. The cDNAs were amplified using gene-specific primers that were designed for the fungal actin genes (Table S5). qRT-PCR experiments were performed with three technical replicates for each sample in 20 µl reaction mixtures containing 2x SYBR Master Mix and 1 µl of 1∶10 diluted cDNA template with gene-specific primers (Table S5) using the LightCycler 480 system and its quantification software (Roche, Basel, Switzerland). By comparing the crossing point (CP) values of the mixed transcriptome samples with those of each artificial control sample, the amounts of fungal RNA were estimated.

### Confirmation of the infection-responsive expression profiles by qRT-PCR

To confirm the infection-responsive expression profiles for the known rice and fungal genes, we performed qRT-PCR experiments using the Transcriptor First-Strand cDNA Synthesis Kit (Roche, Basel, Switzerland), in which 4 µg of the total RNA samples were reverse transcribed in a 20 µl reaction mixture following DNase I treatment. qRT-PCR was also performed using three technical replicates for each gene in 20 µl reaction mixtures containing 2x SYBR Master Mix and 1 µl of 1∶10 diluted cDNA template with gene-specific primers (Table S5) using the LightCycler 480 system and its quantification software (Roche, Basel, Switzerland). The detection threshold cycle for each reaction was normalized against the expression levels of the rice ubiquitin1 and fungal actin genes (Table S5).

## Supporting Information

Figure S1 **Correlation of RPKM distribution between two biological replicates for mixed transcriptome samples.** Comparisons of estimated RPKM distributions between biological replicates for (A) rice compatible, (B) rice incompatible, (C) fungal compatible and (D) fungal incompatible interactions. Pearson's correlation coefficients (*R* ^2^) between replicates and statistical significance levels are presented.
(TIF)

Figure S2 **Cumulative curves of RPKM values for gene expressions of two fungal strains in the conidial conditions.**
(TIF)

Table S1 **Expression profiles and functional annotations of infection-responsive rice annotated transcripts.**
(XLSX)

Table S2 **Expression profiles and functional annotations of infection-responsive fungal annotated transcripts.**
(XLSX)

Table S3 **Expression profiles and functional annotations of infection-responsive rice Cufflinks-predicted unannotated transcripts.**
(XLSX)

Table S4 **Expression profiles and functional annotations of infection-responsive fungal Cufflinks-predicted unannotated transcripts.**
(XLSX)

Table S5 **Primer sequences for RT-PCR experiments.**
(XLSX)

Table S6 **Upregulated rice pathogenesis-related genes.**
(XLSX)

Table S7 **Highly upregulated candidate fungal effectors.**
(XLSX)
