# Supplementary figures and images for: Simultaneous RNA-Seq Analysis of a Mixed Transcriptome of Rice and Blast Fungus Interaction
Source: PLoS One. 2012 Nov 6;7(11):e49423. doi: 10.1371/journal.pone.0049423 (PMC3490861; doi:10.1371/journal.pone.0049423)

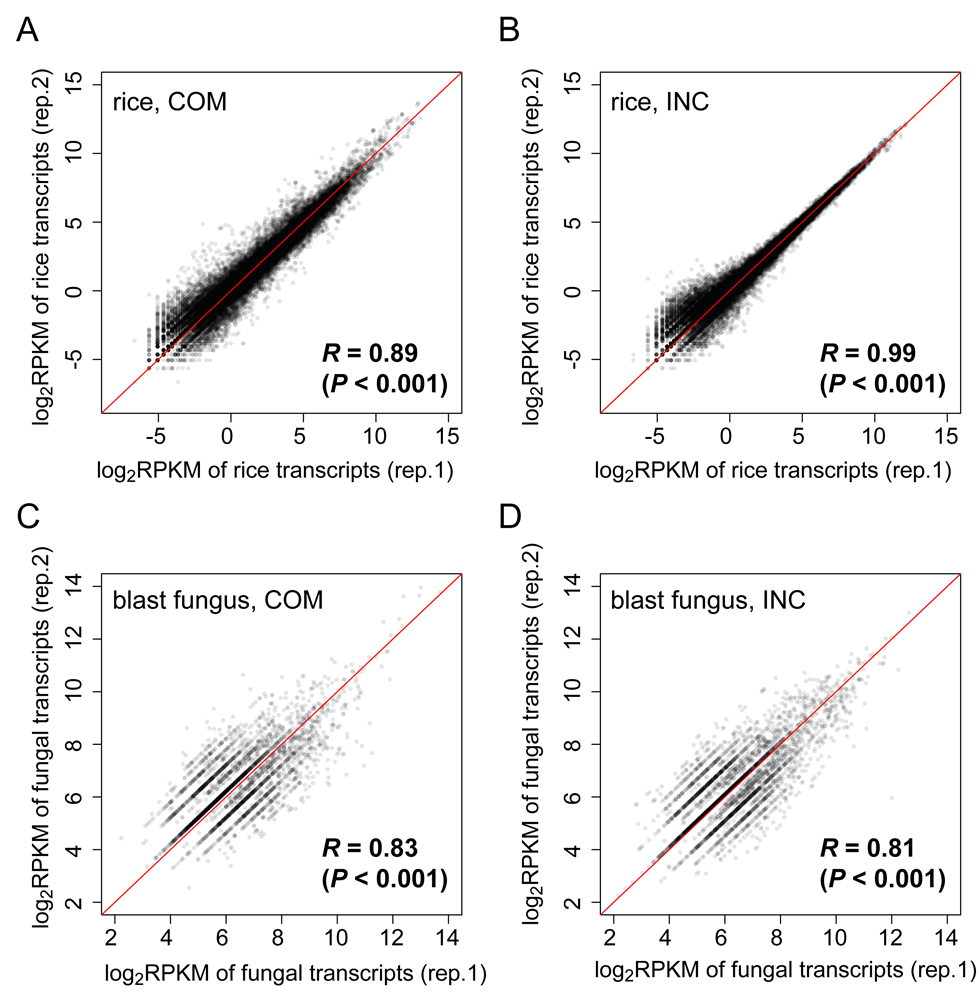

Supplement: Figure S1 — Correlation of RPKM distribution between two biological replicates for mixed transcriptome samples. Comparisons of estimated RPKM distributions between biological replicates for (A) rice compatible, (B) rice incompatible, (C) fungal compatible and (D) fungal incompatible interactions. Pearson's correlation coefficients (R 2) between replicates and statistical significance levels are presented. (TIF) [file pone.0049423.s001.tif]

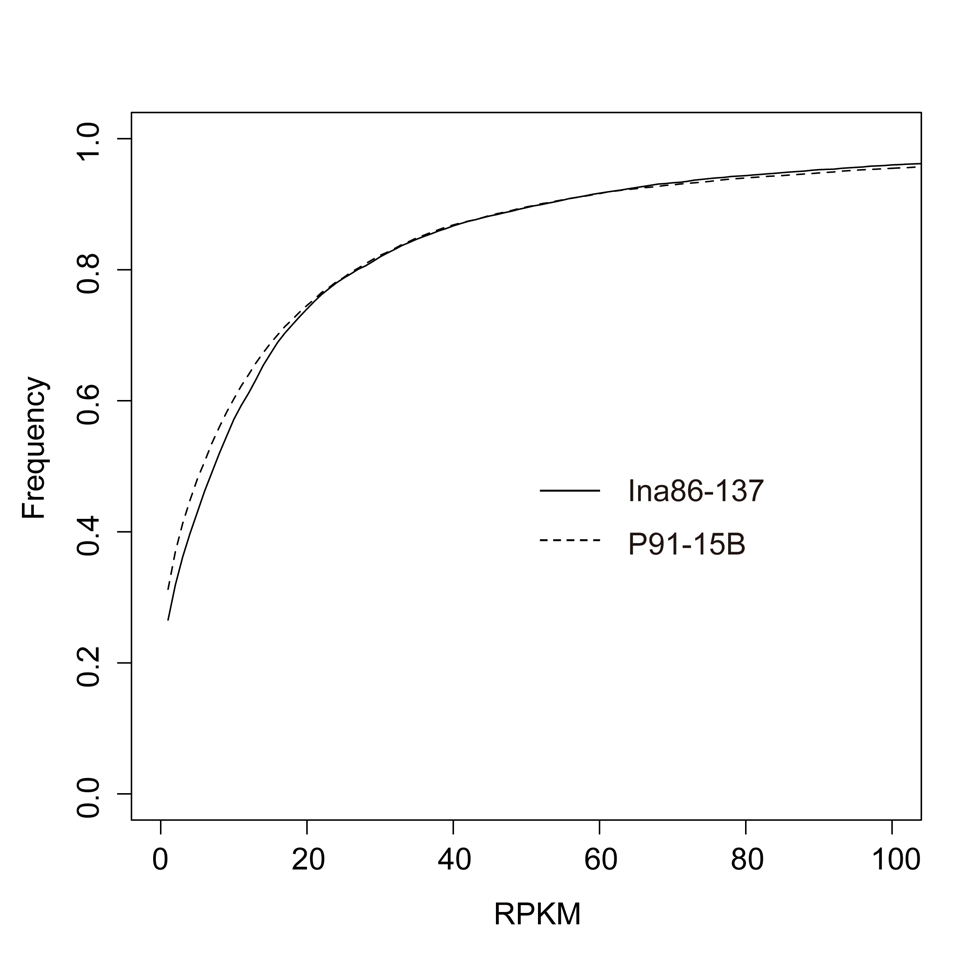

Supplement: Figure S2 — Cumulative curves of RPKM values for gene expressions of two fungal strains in the conidial conditions. (TIF) [file pone.0049423.s002.tif]
